# Polystyrene Opals Responsive to Methanol Vapors

**DOI:** 10.3390/ma11091547

**Published:** 2018-08-28

**Authors:** Luca Burratti, Mauro Casalboni, Fabio De Matteis, Roberto Pizzoferrato, Paolo Prosposito

**Affiliations:** 1 Industrial Engineering Department, University of Rome “Tor Vergata”, Via del Politecnico 1, 00133 Rome, Italy; casalboni@uniroma2.it (M.C.); Fabio.Dematteis@roma2.infn.it (F.D.M.); pizzoferrato@uniroma2.it (R.P.); 2 Centre of Regenerative Medicine, Centre of Regenerative Medicine of University of Rome “Tor Vergata”, Via Montpellier 1, 00133 Rome, Italy; 3 National Interuniversity Consortium of Materials Science and Technology (INSTM), University of Rome “Tor Vergata”, 00133 Rome, Italy

**Keywords:** photonic crystals, methanol vapor sensor, reflectance spectra, capillary condensation

## Abstract

Photonic crystals (PCs) show reflectance spectra depending on the geometrical structure of the crystal, the refractive index (*n_eff_*), and the light incident angle, according to the Bragg-Snell law. Three-dimensional photonic crystals (3D-PCs) composed of polymeric sub-micrometer spheres, are arranged in an ordered face cubic centered (*fcc*) lattice and are good candidates for vapor sensing by exploiting changes of the reflectance spectra. We synthesized high quality polystyrene (PS) 3D-PCs, commonly called opals, with a filling factor *f* near to the ideal value of 0.74 and tested their optical response in the presence of different concentrations of methanol (MeOH) vapor. When methanol was present in the voids of the photonic crystals, the reflectance spectra experienced energy shifts. The concentration of methyl alcohol vapor can be inferred, due to a linear dependence of the reflectance band maximum wavelength as a function of the vapor concentration. We tested the reversibility of the process and the time stability of the system. A limit of detection (LOD) equal to 5% (*v/v*_0_), where *v* was the volume of methanol and *v*_0_ was the total volume of the solution (methanol and water), was estimated. A model related to capillary condensation for intermediate and high methanol concentrations was discussed. Moreover, a swelling process of the PS spheres was invoked to fully understand the unexpected energy shift found for very high methanol content.

## 1. Introduction

Ecosystems, such as air, water, soil, etc., sustain human and animal life providing livelihoods. Environmental pollutants, such as heavy metal ions [1,2,3,4,5,6,7], inorganic, and organic compounds [8,9] are released into the ecosystems, and thus into the entire food chain, producing poisoning effects, which can cause diseases to human and animal beings. A responsible management of resources and waste products can help to maintain habitats and living or working places, with very low levels of contamination [10].

Among organic pollutants, Volatile Organic Compounds (VOCs), which are materials with a high vapor pressure at room temperature, such as ketones, alcohols, ethers, and halocarbons, are very dangerous for human health, especially when they are present in indoor spaces [11,12], where the air exchange is not optimal. The alcohols can cause different deleterious effects on the human body by contact, ingestion, or inhalation. For example, methanol is metabolized by hepatic alcohol dehydrogenase in formic acid, which is one of the major toxic molecules, which can induce death and disability, as well as visual impairment and motoric and cognitive disorders [13,14,15]. Moreover, liquids and vapors of alcohols are highly flammable with obvious risks on work safety [16,17,18]. For all these factors, it is extremely important to be able to detect even very small quantities of VOCs.

Usually, electrochemical sensors able to detect the presence of VOCs, are difficult to produce and are quite expensive. In this case the working principle is based on a change of the conductivity of the material, after exposure to the gas [19,20,21,22]. The development of innovative and low-cost sensors, based on different detection mechanisms is highly demanded. Optical sensors, which have a change of specific optical properties in the presence of contaminants, are a valid alternative to the electrochemical ones, both for the absence of electrical signals, which can be dangerous in specific environments, and for their potential high sensitivity [23,24,25,26,27].

Recently, 1D, 2D, and 3D Photonic Crystals (PCs) have been developed as gas sensors because of their easy preparation and low costs, with respect to other solid-state sensors [28,29,30,31,32,33,34,35]. PCs are sub-micrometer structured dielectric materials with periodic spatial variation of the dielectric function (DF), that produces photonic band gaps or stop gaps [36,37,38,39,40]. As a consequence, a range of visible light wavelengths cannot propagate. In fact, visible light interacting with the PC has a destructive interference and the relative frequencies are reflected [41,42]. The range of wavelengths reflected depends on several characteristics, such as the refractive index (RI) of the material, the geometrical features, and the incidence angle of light. Bragg-Snell’s law states the theoretical behavior of PCs, as described in References [43,44,45,46]. Usually the working principle of PCs sensors is a variation of the RI of the whole system in the presence of a contaminant, which produces an energy shift of the reflectance peak or a change of its shape, according to Bragg-Snell’s law [47]. However, other mechanisms can occur in addition, such as swelling [29,48] or shrinking [49] of the entire PCs, when it is exposed to pollutants.

In this paper, we have synthesized good quality PCs based on commercial polystyrene (PS) nanoparticles, by drop casting on pre-treated glass substrates. The opal films presented a very good reflectivity band with a maximum around 590 nm in air, at normal incidence of light. We studied the time-dependence of the reflectance peak in the presence of different concentrations of methyl alcohol vapors. We mixed methanol and water in liquid state, at different volume ratios, to vary the concentration of alcohol in vapor phase. High values of water vapor concentration inside the measuring chamber did not affect the sensitivity of the PC device significantly, due to the high hydrophobicity of PCs surface, which excludes the penetration of water inside the opal structure. This aspect allowed the detection of methanol vapor, reaching an estimated LOD of our system equal to 5% *v/v*_0_. In this report, we extend previous experiments performed with ethyl alcohol and we used some characterizations and results of that study [29].

## 2. Experimental

### 2.1. Materials

Polystyrene nanospheres (average nominal diameter D = 250 nm) were purchased from Microparticles GmbH (Berlin, Germany). The beads were in a colloidal water solution, with a concentration of 2.5 wt.% The substrates for the sample deposition were commercial *Corning^®^* microscope glass slides (Corning Incorporated, New York, NY, USA) (2.5 cm × 2.5 cm). To have clean and hydrophilic surfaces, substrates were treated with the following procedure: they were first put into H_2_SO_4_/H_2_O_2_ solution (3:1, volume ratio) for 10 min, then in a solution of NH_4_OH/H_2_O_2_/H_2_O (1:0.6:0.8, volume ratios) for 5 min in an ultrasonic bath, and finally in HCl/H_2_O_2_/H_2_O (1:2:7, volume ratios) for 5 min. As a final step, the substrates were washed with deionized water and dried using N_2_ gas. All the chemicals, namely sulfuric acid (98%), hydrogen peroxide (35%, in water solution), ammonium hydroxide (40%), hydrochloric acid (37%), and methanol (99.9%) were purchased from Sigma Aldrich (Milan, Italy).

### 2.2. Synthesis of Polystyrene Photonic Crystals

Microscope glass slides were used as substrates for the PCs synthesis; to increase the structural order of the photonic crystals and to enhance the intensity of reflected light, a circular polymeric mask having a diameter of 17.6 mm was placed onto the glass surface to spatially limit the spreading of the colloidal solution. A volume of 230 µL of the colloidal solution (concentration of 2.5 wt.%) was spread onto the treated glass circular surface. After deposition, samples were dried at 25 °C for 24 h, in an incubator having a controlled humidity (80%). This procedure was necessary to have good PCs with a suitable reflectance and a high mechanical stability.

### 2.3. Apparatus

**Figure 1:**
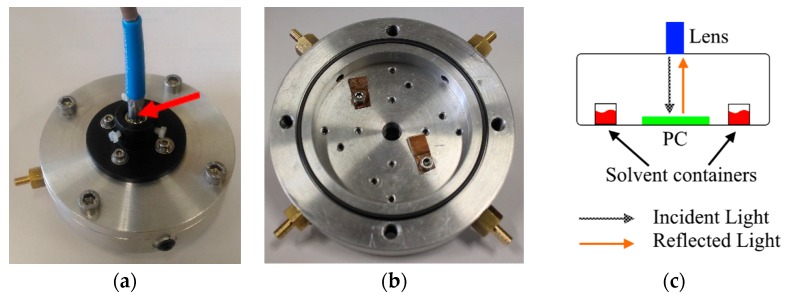
Photographs of the measurement chamber: (**a**) closed chamber, red arrow indicates the specific lens collimating the light source and collecting the reflected light from the PC, the blue part is the optical fiber; (**b**) the opened chamber; and (**c**) a schematic representation of the measurement technique.

Reflectance spectra as a function of incidence angle were recorded by an ellipsometer Wvase 32 (J.A. Woollam, Lincoln, NE, USA) in the range 30°–70°, with respect to the surface normal. The ellipsometer was used as a normal reflectometer (θ_inc_ = θ_ref_), measuring the reflected intensity as a function of the wavelength. For the tests with methanol vapors, we fabricated a closed chamber to perform reflectance measurements at normal incidence. Figure 1a,b report two photographs of our measurement chamber. The sample was fixed on the bottom of the chamber by metal clips to prevent the motion of the PS opal during repeated measurements. The measurement chamber, with a total inner volume of 30.6 mL, was coupled with a halogen lamp (DH-2000, OceanOptics, Largo, FL, USA) through an optical fiber. The fiber enabled the optical excitation by means of a specific lens producing a parallel light beam. The reflected light (normal incidence) from the PC, was collected back by the same lens and sent through a second optical fiber to a compact spectrometer (Flame, OceanOptics, Largo, FL, USA), which allowed for the recording and analysis of the reflected beam. A schematic representation of the chamber and the measurement technique is shown in Figure 1c.

### 2.4. Detection of Methanol

To investigate the change of the optical properties of the PS opal as a function of the environmental conditions, we placed into the measurement chamber, three little containers able to be filled with a total volume of 600 µL. Once the three jars with liquid (blend of methanol/water) had been filled, the chamber was closed and the measurement was started by collecting the reflectance spectra as a function of time (one spectrum every 30 s for 1 h). The first spectrum represented the reference measurement, i.e., the reflectance of the PC in the presence of pure air. As time goes by, the atmosphere inside the chamber saturates with the vapor of the liquid contained in the little containers. The time range of 1 hour was selected, since after the first characterizations, we realized that 60 min were an adequate time to reach a stable condition (no further changes of the reflectance shape for longer times). The effect of the methanol vapor on the measured spectra is a shift of the reflectance peak towards longer wavelengths (red shift), as shown in the next paragraph. To change the percentage of MeOH inside the chamber, we mixed water and methanol with different volume ratios (*v/v*_0_, where *v* was the volume of methanol and *v/v*_0_ was the total volume of the solution).

## 3. Results and Discussion

### 3.1. Optical and Structural Characterization of PS Opals

**Figure 2:**
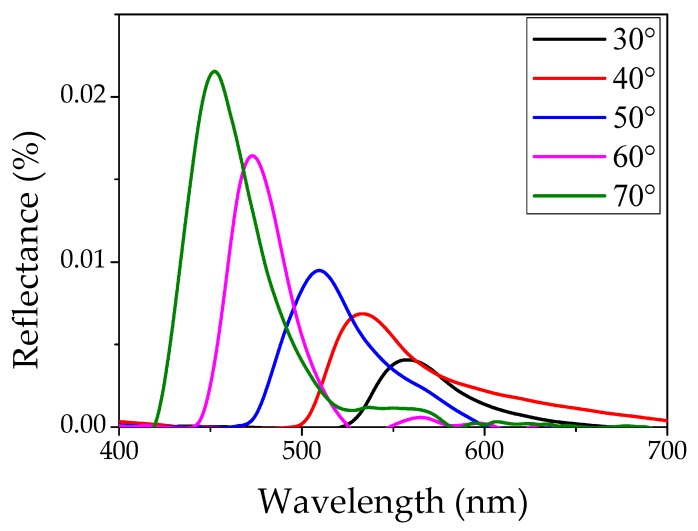
Reflectance spectra experimentally measured of polystyrene (PS) opal as a function of light incidence angle.

In this paragraph, we will characterize the opal structure and verify its operating principle. In general, the Bragg-Snell’s law describes the behavior of a photonic crystal, where the geometrical structure, the mean diameter of particles, and the effective refractive index are fundamental factors [43,47]. The formula giving the wavelength of maximum refection is:

(1) $${m\lambda \;}_{max}=2\;\sqrt{\frac{2}{3}}D\;\sqrt{{(fn}_{p}^{2}\;+\;\left(1\;-\;f\right)n_{m}^{2}{)\;-\;sin}^{2}\theta }$$

where *m* is the diffraction order, *λ_max_* is the wavelength of the maximum of the diffraction peak, *D* is the diameter of the nanospheres, *θ* is the incident angle of light, *n_p_* and *n_m_* are the refractive indexes of the particles and of the material between the dielectric spheres (generally air), respectively. Finally, *f* is the volume fraction (known as filling factor) of the particles in *fcc* structure (for an ideal lattice *f* is 0.74). The first term inside the brackets under the square root, represents the effective refractive index (*n^2^_eff_*) of the whole system. Knowing the physical variables listed above, it is possible to calculate the filling factor (*f*) by a fitting procedure of the diffraction peak wavelengths at different incident angles, as in Reference [29]. In our case a value of 0.73 was estimated indicating the good quality of the system. Figure 2 shows the reflectance spectra at specific angles (from 30° to 70°); the maximum shifts to shorter wavelengths and the intensity increases with the increase of the angle of reflection. In previous work [29], we determined a very good correlation between the fitting of the optical data and SEM (Scanning Electron Microscopy, Leo Supra 35, Zeiss, Jena, Germany) images (not shown here), confirming the high quality of our PS opals.

### 3.2. Optical Behavior of PS Opal in the Presence of Different Methanol Concentrations

**Figure 3:**
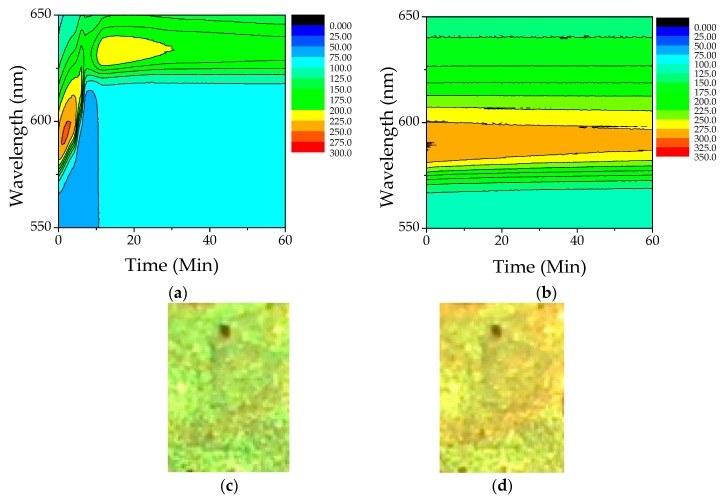
Color maps of the reflectance spectra as a function of time: (**a**) pure alcohol and (**b**) pure water (0% *v/v*_0_ methanol). Colors are proportional to the intensity (counts) of the reflected signal. Photographs of the sample surface without methanol (**c**) and after one hour of exposition to methanol vapor (**d**).

To guarantee a stable temperature of 23 °C, the measurement chamber was kept in a water refrigerator system. We studied the reflectance of PS photonic crystals, at normal incidence (Θ = 0°), in the presence of different concentrations of methanol/water vapors. Figure 3a shows the color map of the reflectance peak as a function of time, for the case of pure methanol. In this case, the maximum red shift was about 48 nm and was reached in a very short time (about 10 min). The intensity of the reflectance band (color scale in the figure), varied up to a plateau value after 30 min. The initial increase in reflectance could probably be related to the formation of a thin layer of condensed methanol onto the PC surface. A similar analysis was also performed for pure water, to test the response of the optical sensor in presence of high levels of humidity and to understand if water can produce a variation of the reflected signal. Figure 3b reports the reflectance maximum as a function of time, for pure water. Figure 3c shows the photograph of the sample surface before the exposition to methanol vapor, whilst Figure 3d shows the photograph of the same point, after one hour of exposition to methanol vapor. A change of color is evident.

Table 1 shows the investigated concentrations of methanol in liquid and in gas phases [29]. To calculate the vapor pressure of methanol and water we used Antoine’s law, and by means of Raoult’s law, we estimated the partial pressures of methanol vapor, water vapor, and the air in the chamber [29]. From ideal gas law, we estimated the methanol vapor mole fraction, and the corresponding ppm concentration. The mechanism inside the measurement chamber was the following: after closing the chamber, the liquid solvent inside the little trays started evaporating and saturated the small volume inside the chamber. Subsequently, the vapor could condense on all the internal surfaces of the chamber, and on the top of the PC surface. Due to the porosity of the PC, the liquid on the crystal surface could easily penetrate the inner layers of the PC. This hypothesis was strongly supported by naked eye observation, especially for the pure methanol case. In this case, when we opened the experimental apparatus, an evident wet layer of the solvent covering both the chamber and the PS opal surface was clearly visible.

**Figure 4:**
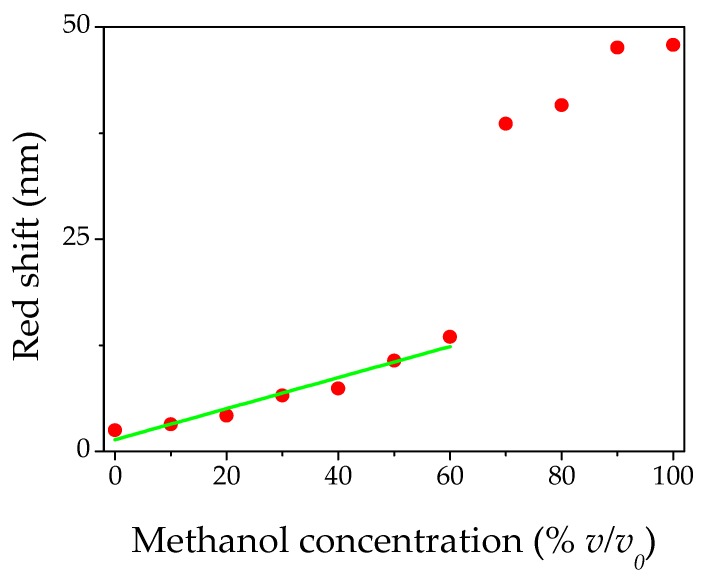
Red shift as a function of methanol concentration. The green line represents the fitting of linear behavior of sensor.

Figure 4 shows the red shift behavior of the reflectance peak as a function of the relative methanol percentage in solution. The main process responsible for the change in the red shift in the photonic crystal theory, is related to a change of the effective refractive index. The general dependence [43,47] of *n_eff_* for a photonic crystal made of PS spheres can be written as:

(2) $$n_{eff}=\sqrt{0.74\;n_{p}^{2}\;+\;0.26\;n_{pores}^{2}}$$

where *n_p_* and *n_pores_* are the refractive indexes of PS particles and the pores between the organized spheres, respectively. The constants 0.74 and 0.26 are the volume fractions of the PS spheres and the voids, respectively.

**Figure 5:**
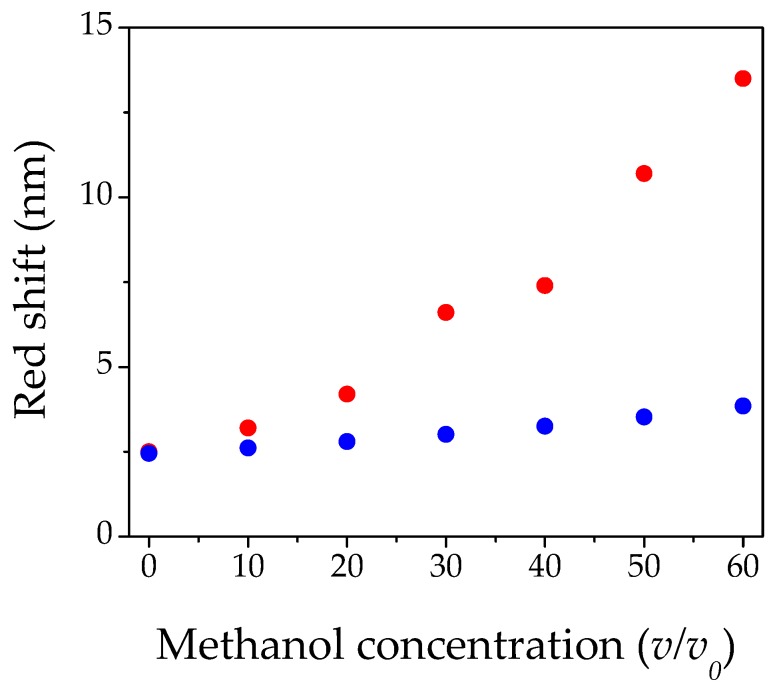
Red shift as a function of methyl alcohol concentration: blue circles are the calculated red shift, while red circles are the measured red shifts.

In our case the simultaneous presence of methanol, water, and air made the formula slightly more complicated. It could be written as:

(3) $$n_{eff\;}=\sqrt{{0.73\;n}_{p}^{2}{\;+\;t\;n}_{MeOH}^{2}{\;+\;g\;n}_{water}^{2}\;+\;\left(0.27\;-\;t\;-\;g\right)n_{air}^{2}}$$

where 0.73 was the actual filling factor of our photonic crystal, and *n_p_*, *n_MeOH_*, *n_water_*, and *n_air_* were the refractive indexes of PS particles, methanol, water, and air, respectively. The factors *t* and *g* were the volume percentages of methanol and water in vapor form obtained, considering the molar fraction of the respective vapors. These factors are mainly proportional to the partial vapor pressure of methyl alcohol and water inside the measurement chamber. We took into account the ideal behavior of the gas and liquid phase. If we consider the refractive index of the methanol and water vapor phase in the equation, the refractive index remains almost constant, independently of their concentrations, and the red shift remains fixed at zero (no variation with respect to pure air). This behavior is totally in disagreement with our experimental results. On the other hand, if we consider a condensation process of the vapors proportional to partial vapor pressure of methyl alcohol and water inside the measurement chamber, we can use the refractive index of the liquids, instead of those of the vapors in Equation (3). Figure 5 shows the calculated trend (blue circles) obtained with this approximation, with respect to the experimental behavior (red circles). Even in this case, a strong disagreement can be noticed. A gradual deviation from the calculated trend was observed, for concentrations greater than 20%. We ascribed such a difference to an enhanced capillary condensation process, induced by the nanopores of the PC. The phenomenon of capillary condensation, consists in a phase transition from vapor to liquid state, for pressures even lower than the saturation value of a certain solvent in mesoporous materials [50,51,52,53,54], such as our opals. For this concentration range, PS pores are filled by liquid solvents in a much higher proportion with respect to the standard condensation, and the effective refractive index of the system is higher than the calculated one.

**Figure 6:**
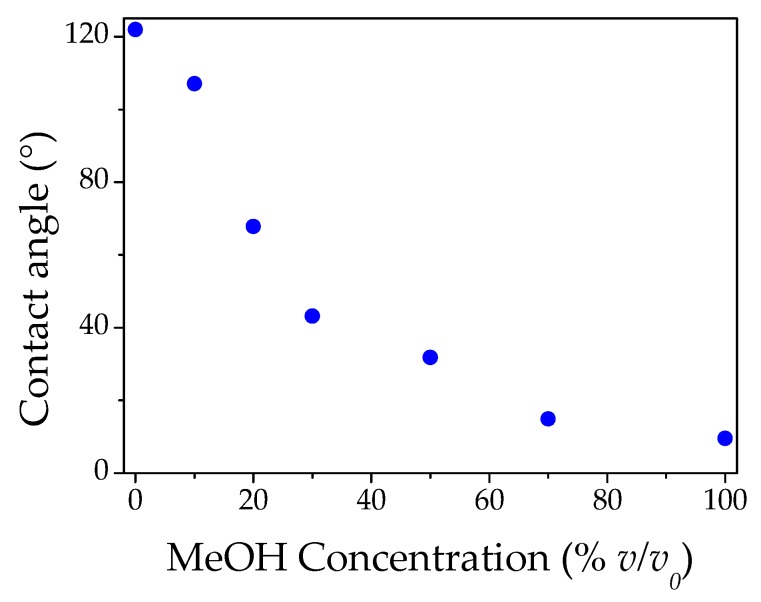
Contact angles as a function of methanol content.

**Figure 7:**
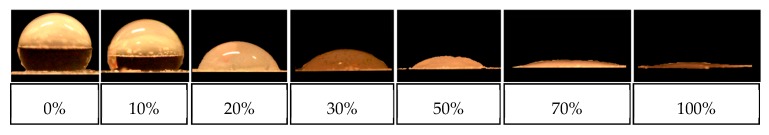
Photographs of droplets of methanol/water solutions with different volume ratios (*v/v*_0_) indicated in each picture.

Following this hypothesis, the first remark regards the small red shift value at 0% methanol concentration (pure water). If the capillary condensation was the driving force of the mechanism, we would have expected an intense red shift related to the complete filling of the pores with water. However, this did not occur since the PC surface was highly hydrophobic, due to the nanostructured surface and the polystyrene material. In this regard, we performed contact angle characterization of the PC surface. As shown in Figure 6, the contact angle changes considerably as a function of the different concentrations of MeOH/H_2_O. For pure water, the PC surface presented a very high contact angle, indicating a high level of hydrophobicity. Nevertheless, with increasing concentration of methanol in water, the contact angle decreased, conferring a higher wettability to the PC surface. This important characteristic strongly influences the infiltration of the condensed solvent vapor inside the opal apertures: the higher the methanol concentration, the higher the surface wettability and infiltration inside the PC pores. Figure 7 shows photographs of a small drop of mixtures MeOH/H_2_O, at the different concentrations reported in the figure. The difference in the drop shape indicated the hydrophobic/hydrophilic behavior of the PC surface, as a function of the drop composition. Contact angles greater than 90° define a surface as hydrophobic. In this case, the capillary condensation is strongly hampered. The small red shift measured at 0% methanol, could therefore be inferred to a thin layer of water on the surface, which can penetrate only into the first few layers of the PC, changing the reflectance maximum wavelength of about 2.5 nm. Increasing gradually, the methanol percentage up to 60%, the partial pressure of methanol increases, and the capillary condensation process becomes more effective and fills the pores with liquid methanol proportionally to the percentage of the methanol/water blends.

The presented model could justify the experimental results, up to 60% *v/v*_0_. For higher concentrations, a very intense red shift is detected, and an additional mechanism must be considered. In this case, the methanol concentrations are so high that the saturation vapor pressure is reached within very short times (within 10 min), and therefore the alcohol condenses almost immediately onto the PC surface and inside the pores. For these concentrations, the presence of water can be neglected for at least three reasons. First, the amount of water in solution is very low. Secondly, the vapor pressure of pure water is several times lower than methanol, thus methyl alcohol will saturate the whole chamber more quickly than water. Finally, the hydrophobicity of the PC surface prevents the penetration of the residual liquid water. In this case, the measured red shift (about 48 nm) is not compatible with just the complete filling of the pores with liquid methanol, which would give a maximum value of 30 nm, but a swelling process of the PC spheres has to be considered, as already proposed in a recent paper [29].

**Figure 8:**
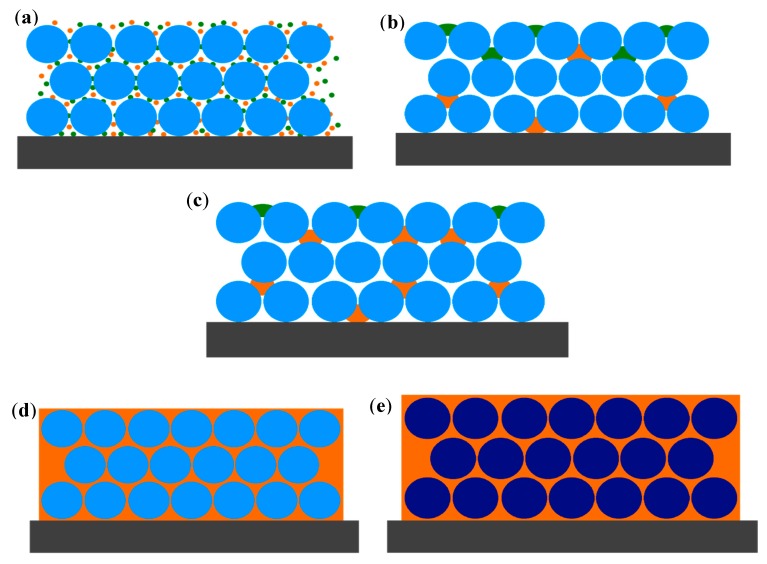
Schematic representation at the vapor saturation condition, five processes are described: (**a**) methyl alcohol and water remain in vapor state; (**b**) MeOH and water vapors partially condense, above and between PS nanobeads; (**c**) methanol vapor condenses in a more efficient way, due to the capillary condensation; (**d**) MeOH vapors condense completely inside the PC; and (**e**) MeOH vapors condense and swell PS particles.

Figure 8 shows a schematic drawing of the PC behavior in the presence of methanol, water, and air. Five possible scenarios are shown, where the grey rectangles represent the glass substrates, light blue circles are PS nanospheres, and dark blue circles the swollen PS nanobeads. Orange points are molecules of methanol in gas phase and the orange background is MeOH in liquid form. Green points are molecules of water in vapor phase and the green zone is water in liquid state. In the first case (Figure 8a), methanol and water remained in the vapor phase, as mentioned above. In this case, the effective refractive index is expected to remain almost constant and the reflectance spectrum will be the same as in the case of pure air. Figure 8b shows the possible picture for small methanol concentration (0–20%). A very small condensation of water (for the high hydrophobicity of the surface), and a partial condensation of the methanol fills the pores, inducing only a small red shift. Figure 8c represents the situation for methanol concentrations up to 60%, in this case, the liquid MeOH gradually fills the pores and an increase of *n_eff_* is expected together with a red shift of the reflectance peak. In the fourth picture (Figure 8d), methanol vapors condense completely and penetrate inside spaces of PS particles, saturating all voids. Water (green zone) is not reported anymore. In this case, *n_eff_* will be given only by the two contributions of polystyrene particles and methanol, according to Formula (3). Replacing this effective refractive index in Equation (1), we can predict a value of *λ_max_,* corresponding to a red shift of about 30 nm, which is however, lower than that obtained by our experimental data. In the last representation (Figure 8e), in addition to a complete filling of PC voids by liquid solvent, also a swelling of the PS beads is considered, producing a little increase of the mean diameter of the spheres. In this case, referring to Formula (1), the parameter D changes to D’ (swelled diameter), with D’ > D. This hypothesis was confirmed by an independent measurement of the swelling properties of methanol on polystyrene, where we estimated a weight swelling of 3.3% [29]. Considering the swelling process, which induced an average increase of the sphere diameter of about 3%, our experimental results were fully justified.

Regarding the gap in the red shift of Figure 4, between 60% and 70% *v/v*_0_, we could suppose that it is the result of the two different competing phenomena described above. For low concentrations, capillary condensation can occur for a lower value of the partial pressure, whilst for a high solvent percentage the condensation certainly occurs and fills completely the PC pores, also inducing a swelling of the material. A similar behavior was reported by Zheng et al., for ethanol/water blends [55]. Despite the presence of water vapor inside the measurement chamber, we estimated the limit of detection (LOD) of our optical device to be 5% *v/v*_0_. This result was due to the combined action of the intrinsic hydrophobicity of PS and the hydrophobic structured surface of the opal.

## 4. Conclusions

We developed a photonic crystal based on self-assembled polystyrene nanospheres, which shows an optical response to methanol vapors. This system was able to detect the presence of different concentrations of methanol by a change of the reflectance spectrum, induced by the condensation of the vapors inside the pores of the PS opal. In particular, we detected a wavelength red shift of the reflectance peak, due to the increase of the effective refractive index of the entire system and to the swelling of PS spheres induced by the alcohol. Such PCs were not affected by the presence of water, due to the properties of the material and to the surface nanostructuring, which prevented the infiltration of water inside the pores. Exploiting these properties, we tested its response to a mixture of two substances, water and methanol. We proposed a model for the behavior at methanol concentrations lower than 60% *v/v*_0_, based on partial capillary condensation. On the other hand, for high methanol content, a complete condensation and a swelling process were considered. In summary, PS photonic crystals demonstrated to be responsive to methyl alcohol vapors for various water/methanol solutions.

## Figures and Tables

**Table 1:** Methanol concentrations in liquid phase and in vapor phase inside the measurement chamber.

Liquid Phase MeOH:H_2_O (vol:vol)	Percentage of MeOH in Liquid Phase	MeOH in Liquid Phase (Molar Fraction)	MeOH in Vapor Phase (Molar Fraction)	MeOH in Vapor Phase (ppm)
1:9	10%	0.047	0.070	7843
1:4	20%	0.100	0.015	16,669
1:2.3	30%	0.160	0.024	26,627
1:1.5	40%	0.229	0.034	37,966
1:1	50%	0.308	0.046	50,998
1.5:1	60%	0.400	0.060	66,130
2.3:1	70%	0.509	0.076	83,915
4:1	80%	0.640	0.096	105,117
9:1	90%	0.800	0.119	130,828
10:0	100%	1.000	0.149	162,655
